# Targeting PI3Kδ in cancer: a setback or the end

**DOI:** 10.3389/fonc.2025.1614882

**Published:** 2025-11-03

**Authors:** Zhonglin Hao, Susanne Arnold, Jill Kolesar

**Affiliations:** ^1^ Markey Cancer Center and Department of Internal Medicine, University of Kentucky, Lexington, KY, United States; ^2^ Holden Cancer Center and School of Pharmacy, University of Iowa, Iowa City, IA, United States

**Keywords:** phosphatidylinositol 3-kinase delta inhibitors, indolent lymphoma, adverse events, intermittent dosing, solid tumor immunotherapy

## 1 Overview

The phosphoinositide 3-kinase delta (PI3Kδ) is a member of the class I lipid kinase family based on its sequence homology in kinase domains and its substrate selectivity (1, 2). First isolated from a cDNA library made from monocytes, the PI3Kδ gene is selectively expressed in leukocytes. Signaling through this kinase is via cytokine receptors (3, 4). Subsequent functional studies revealed impaired B and T cell antigen receptor signaling in mouse models which have a nonfunctional kinase allele (4). Development of pan-kinase and delta selective inhibitors marked the beginning of a new era in the treatment of B cell malignancy. Meanwhile, the role of PI3Kδ in Treg function prompted exploration of it as a tool for breaking the immune tolerance to boost antitumor immunotherapy. The science behind the translational efforts was captured in this review (5). Unfortunately, clinical use of this class of drug in management of B cell malignancies and research came to halt when all FDA approvals of this drug class were withdrawn (6). Here, we discuss the initial approval of four PI3K inhibitors. We present preclinical studies supporting use of the inhibitor in modulating the tumor immune microenvironment (TIME) to boost tumor immunity. We highlight the challenges of using such inhibitors in the clinic that led to withdrawal of this class of PI3Kδ inhibitors in hematologic malignancy. Finally, we propose strategies to mitigate the adverse effects while retaining the efficacy in future clinical trials, highlighting potential areas of further exploration. We conclude that exploration of PI3Ki will continue despite this recent setback.

## 2 Approval and withdrawal of PI3Kδ targeted inhibitors in B cell malignancies

PI3K inhibitors conferred high overall response rate between 57-74% and improvement in progression free survival between 11.0 to 21.5 months in single agent treatment or combination settings. The high response rate was linked to their ability to drive B cells out of their protective lymph nodes or spleen niche (section 3 below). Long term observation using overall survival data however, failed to show advantage over the control arms. The dosing schedules were associated with high incidence of adverse events.

Idelalisib is an orally bioavailable ATP-competitive kinase inhibitor given continuously that targets the PI3K p110 isoform δ (PI3Kδ) with high potency and selectivity. In an open labeled single arm phase 2 study, 125 patients with indolent lymphoma who had not responded to rituximab or alkylating agents or who had recurrence after 6 months were given 150 mg of drug twice daily until disease progression or withdrawal. Overall response rate (ORR), which was the primary end point, was 57% with 6% complete response. Median progression-free survival (PFS) was 11 months. Severe adverse events included neutropenia (27%), increased liver function tests (13%), diarrhea (13%) and pneumonia (7%). As the first-in-class drug, idelalisib’s initial approval for B cell malignancies in July 2014 included indications in relapsed/recurrent CLL, SLL and follicular lymphoma (FL) (7). The approval in FL and in SLL was an accelerated one that mandated post-marketing trials which suffered from slow accrual. The regular approval was then noted to have a death rate due to drug toxicity more than double that of the control arm in a randomized trial in 2016 and FDA mandated a box warning on the label (8). The sponsor voluntarily withdrew idelalisib from the market in 2022 due to inability to complete the confirmation study.

Copanlisib, which is considered a pan-PI3K inhibitor, received accelerated approval in 2017 for relapsed FL. The approval was based on CHRONOS-3, a multicenter, randomized, double blinded, placebo-controlled phase 3 trial of 652 patients with CD20 positive indolent lymphoma which recurred 6–12 months before the last dose of rituximab. Patients were randomized into receiving either copanlisib and rituximab or rituximab and placebo at a 3:1 ratio and were observed for PFS. The drug was given intravenously on days 1, 8, and 15 every 4 weeks. The trial showed a PFS advantage of 21.5 months with copanlisib plus rituximab over rituximab plus placebo (13.8 months). The most common serious adverse events (SAEs) were hyperglycemia (56% vs 8%) and hypertension (40% vs 9%). The drug was well tolerated overall with only one death from pneumonitis (9). Interim analysis, however, showed no overall survival (OS) advantage. In 2021, the agreement between the FDA and the drug maker was voluntarily withdrawn based on CHRONOS-3 results pending new study results from the ongoing CHRONOS-4. Unfortunately, CHRONOS-4 was not a positive study in relapsed indolent lymphoma showing no PFS or OS benefit in patients receiving rituximab and bendamustine plus copanlisib compared to rituximab and bendamustine plus placebo (32.9 m vs 33.3 m; HR: 1.13; 95% CI: 0.80–1.60; P = 0.71) (10). Indeed, the curve of OS showed potential harm in patients receiving copanlisib compared with placebo. Copanlisib was voluntarily withdrawn from the market for having no efficacy.

Duvelisib is a dual inhibitor of PI3Kδ, γ developed for treatment of hematologic malignancies. In 2018, the FDA approved durvelisib in relapsed/refractory (R/R) CLL/SLL after two or more lines of treatment based on the DUO trial (11). The DUO trial is a global phase 3 trial comparing the efficacy of monotherapy for relapsed/refractory CLL/SLL with duvelisib (25 mg twice a day orally) or ofatuzumab. Patients were randomized in a 1:1 ratio into either treatment. The primary end point, PFS, was 13.3 months for duvelisib versus 9.9 months for ofatumumab (HR: 0.52; 95% CI: 0.39–0.69; P<0.0001). The most common adverse events were diarrhea, neutropenia, pyrexia, anemia and cough. The ORR was significantly higher in the duvelisib arm vs ofatumumab (74% vs 45%). The final analysis, however, showed no OS benefit (HR: 1.09; 95% CI: 0.79–1.51). The duvelisib approval in R/R FL in 2018 was an accelerated one based on the response rates at 6 and 12 months and the approval mandated additional post marketing trial which never occurred. Finally, the approval in FL was voluntarily withdrawn in 2022. Further analysis of the DUO trial’s OS showed that the treatment arm crossed over to show potentially more death after ~45 months compared with the ofatumumab (HR: 1.06; 95% CI: 0.71–1.58).

Umbralisib is a dual inhibitor of PI3Kδ/casein kinase-1ϵ, thought to exhibit improved selectivity for PI3Kδ compared with other PI3K inhibitors. In a phase IIb open labeled multi cohort trial enrolling 208 patients designed to evaluate the efficacy and safety of umbralisib in patients with R/R indolent non-Hodgkin’s lymphoma who are CD20+ and not responding to at least one anti-CD20 treatment. Patients were given umbralisib 800 mg orally once a day until disease progression, unacceptable toxicity or withdrawal. The ORR was 47.1% and tumor reduction occurred in 86.4% of patients. The median time to response was 2.7–4.6 months. The median duration of response was not reached for marginal zone lymphoma (MZL), 11.1 months for FL, and 18.3 months for SLL. Median PFS was not reached for MZL, 10.6 months for FL, and 20.9 months for SLL. At least one serious AE occurred in 53.4% of patients and included neutropenia (11.5%), diarrhea (10.1%) and increased LFTs (~7%) with 31 patients discontinuing study due to SAEs (12). The FDA gave accelerated approval to umbralisib for patients with R/E (extranodal) FL who received three or more lines of treatment, or R/R MZL who had received one or more lines of anti-CD20 treatment. In a phase 3 trial comparing umbralisib plus ublituximab versus obinutuzumab and chlorambucil in untreated R/R CLL, the PFS was improved with HR 0.55 (13). However, interim analysis showed that OS was worse with HR 1.23 (14). The manufacturer eventually withdrew all applications from CLL, SLL, FL and MZL due to lack of OS benefit.

## 3 Use of PI3K inhibitors as a tool to target Treg in the TIME

**Figure 1:**
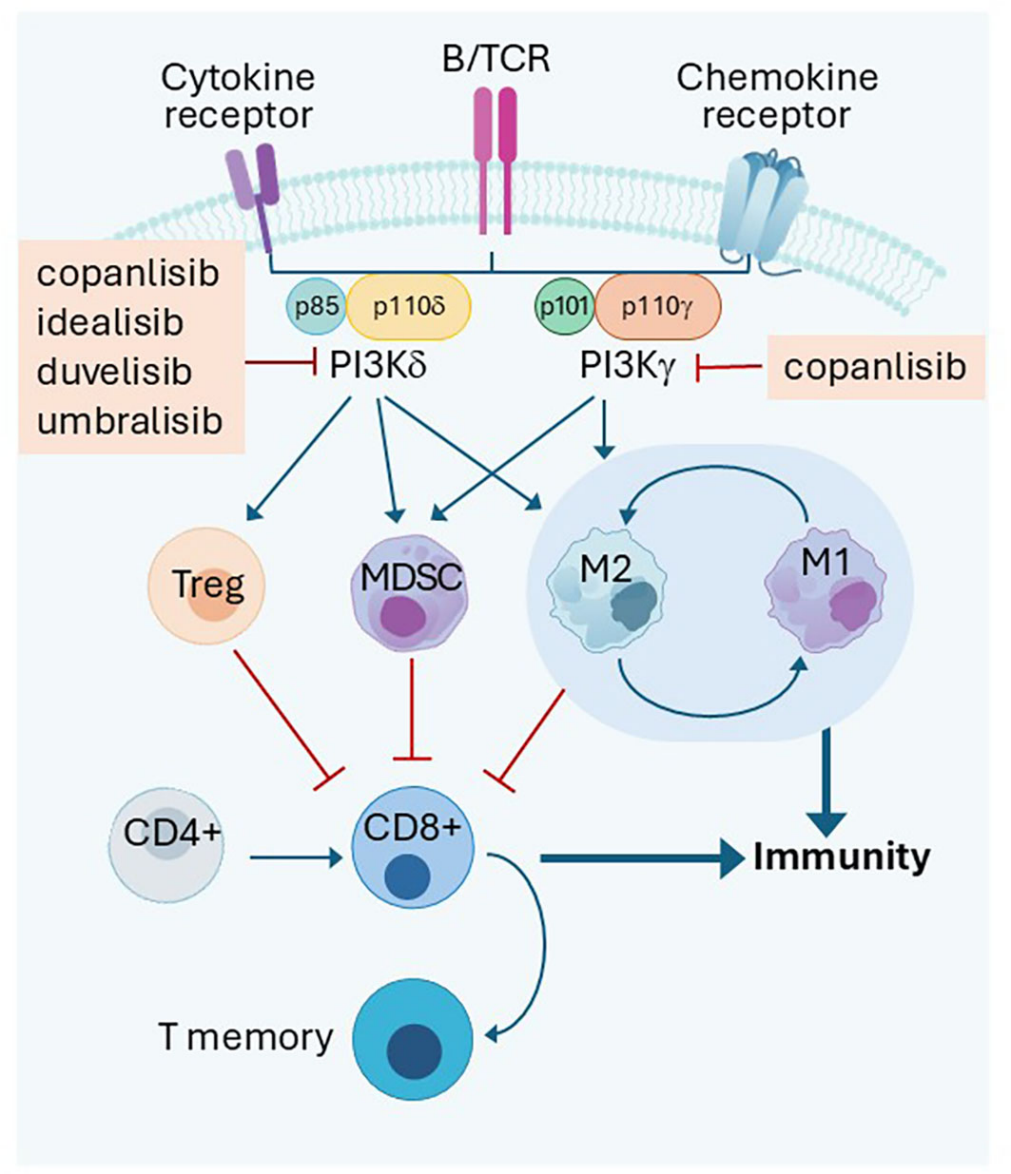
PI3Kδ and PI3Kγ dependent immune cell interactions in the tumor microenvironment. PI3Kδ or PI3Kγ transduce signal inside Treg to keep autoimmunity under check in normal tissue. However, it is a mechanism used by cancer in the tumor microenvirenoment to inhibit tumor immune response along with MDSC and macrophages. Inhibition of both kinases activate immunity. B/TCR: B or T cell receptors Treg: T regulatory cells; MDSC: Myeloid derived suppressor cells M1:M1 Macrophages, inflammatory; M2:M2 macrophages, immune suppressive Promotion 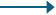
Inhibition 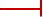.

PI3Ks phosphorylate the inositol ring of phosphatidylinositol (PtdIns) lipids. Three classes are documented, and Class I is noted for its role in regulating cell signaling at and downstream of the plasma membrane. Class I PI3Ks consist of a regulatory subunit (p85) and a catalytic subunit (p110), which exists in four isoforms: α, β, γ, and δ. Whereas p110α and p110β are ubiquitously expressed in different tissues, p110γ and p110δ are mainly concentrated in leukocytes. Widespread high throughput sequencing discovered that p110α mutations were present in 14% of all solid tumors, strengthening the belief that targeting PI3Kα in solid tumors is sound (15). Initial successes in the clinics targeting PI3K in B cell malignancies using delta selective idelalisib ushered in an era of subtype targeting. The impact of different inhibitors on PI3Kδ and PI3Kα function is studied in BaF3 cells (16). It is noteworthy that Treg downregulation by PI3Kδ inhibitor emerged as an approach of TIME manipulation (**Figure 1**).

### 3.1 PI3Kpathway is essential for Treg function

PI3Kδ is critical for transmission of signals from B/T cell receptors. The critical role of PI3Kδ in maintaining Treg proliferation and immunosuppressive function toward T conventional cells was demonstrated in 2006 (17). Tregs with non-functional PI3Kδ (D910A/D910A) had attenuated suppression function and failed to protect mice against experimental colitis in an adoptive T cell transfer. Further work showed that inactivation of PI3Kδ in mice protected them against formation of a broad range of solid tumors as well as hematological malignancy engraftment. Inactivation of PI3Kδ in Tregs genetically or pharmacologically achieved the same purpose (18). PI3K-AKT is essential for *in vivo* maintenance of murine Tregs. Genetic inactivation of PI3Kδ D910A/D910A in a murine chronic lymphocytic leukemia (CLL) model resulted in defective Treg expansion and B cell receptor signaling. The TIME protected the mice from acquiring CLL and acute myeloid leukemia (AML). Reconstitution of the D910A/D910A mice with wild type reversed the leukemia resistance (19). PI3Kδ inhibition likely unleashed a potent T cell-mediated anti-tumor immune response. Inhibition of PI3Kδ in human T cells selectively inhibits Treg proliferation compared to T conventional cells. Inhibition of PI3Kδ also slows tumor growth (20). Human Tregs are highly sensitive to PI3Kδ specific inhibition. Idelalisib specifically inhibited human Tregs and was 4–13-fold more potent in inhibiting Treg than conventional CD4 and CD8 T cells (21). PI3Kδ has a crucial role in shaping the TIME, especially the suppressive cells in head and neck cancer. PTEN loss and PI3Kδ activation was linked to weakened expression of MHCI and MHCII when induced by IFNγ, while PI3Kδ inhibitor was able to enhance it (22). Signal transduction via PI3Kδ and its impact on immune cell function is illustrated in **Figure 1**.

Consistent with a role of upregulation of autoimmunity by PI3K inhibitors, prolonged heavy suppression of PI3Kδ in Tregs is considered responsible for a subset of the adverse effects associated with PI3Kδ inhibitors approved and then withdrawn from the market which include, among others, colitis, hepatitis and pneumonitis (6).

### 3.2 Treg cells suppress cytotoxic T cell function

In early adoptive T cell transfer experiments, transfer of CD4+CD25-Th cells with tumor/self-reactive CD8+ T cells into CD4+ T cell-deficient hosts induced autoimmunity and regression of established melanoma. Transfer of Treg cells (phenotypically CD4+CD25+Foxp3+) was able to block autoimmunity and the efficacy of adoptive T cell immunotherapy. These findings reveal that the presence of naturally occurring Treg cells prevents Th cells from breaking down tolerance to persisting self-antigens and treatment of established tumors (23). Treg cells have become known for their crucial role in the regulation of cancer immunity (24).

### 3.3 Treg Infiltration is prognostic of survival in solid tumors

In ovarian cancer, recruitment of Tregs into the tumor was suspected to foster immune privilege. Higher Treg infiltration was associated with reduced overall survival (25). In hepatocellular carcinoma, increased Tregs impairs CD8+ T cell function and correlates with poor survival (26), while higher Treg infiltration seems to correlate with higher tumor burden and advanced stage in lung cancer (27). In gliomas, Treg tumor infiltration prefers the astrocyte lineage and higher grade tumors such as glioblastoma multiforme, although its prognostic value in survival was not demonstrated (28). In immunogenic tumors such as lung cancer, direct immunosuppression from Tregs, myeloid derived suppressor cells (MDSCs) and tumor associated macrophages is one of the important mechanisms of tolerance causing the tumor to either not respond to treatment from the beginning or to lose response to immune checkpoint inhibitors (ICI) overtime. Increased Tregs are linked to immune escape by dampening the CD8+ T cell tumor infiltration and cytotoxicity (29). In a systemic review and meta-analysis of 76 papers covering 15,512 cases encompassing 17 types of cancer, high Treg infiltration was found to be associated with poor overall survival (OS), with an odds ratio of 1.46 (P<0.001). This effect is particularly evident in immunogenic tumors such as cervical, renal, lung and melanoma which are among the cancers for which ICI received early approval (30). Clinically, increased circulating myeloid-derived suppressor cells (MDSCs) has been associated with poor response to anti-CTLA4 therapies in melanoma patients (31).

### 3.4 Targeting PI3K signaling improves cancer therapy

PI3K inhibition with low-dose duvelisib improved T cell activation resulting in improved T cell-mediated cytotoxicity when added to ICI in an animal model by inhibiting MDSCs (32). Adding the PI3K eganelisib broke the tolerance of the tumor model of 4T1 (breast) and B16 (melanoma) to combination anti-PD-1 and anti-CTLA4 resulting in complete remission of tumors in 30% and 80% of the animals, respectively (33). In a 4T1 breast cancer model, PI3K facilitated tumor growth and inhibited tumor immune surveillance. Genetic deletion of *PI3Kγ* or pharmacological inhibition could partially attenuate the effects. Treatment with a pan Class I PI3K inhibitor and ICI resulted in consistent inhibition of tumor growth compared with either agent alone (34).

Intermittent dosing with copanlisib (pan inhibitor of PI3K) *in vivo* resulted in strong anti-tumor efficacy. Copanlisib increased tumor infiltration of activated T cells and macrophages, and increased CD8+ T cell/regulatory T cell and *M1/M2* macrophage ratios. The combination of intermittently dosed copanlisib with the ICI antibody anti-PD-1 demonstrated enhanced anti-tumor efficacy in both ICI-sensitive and resistant mouse models. In an ICI-sensitive model, combination therapy resulted in complete remission and prevented tumor recurrence (35). An intermittent schedule of the PI3Kα/β/δ inhibitor, BAY1082439, overcame ICI resistance in PTEN-null prostate cancer models. It is not clear however, inhibition of one or all subunits contributed to this effect. BAY1082439 promoted clonal expansion of tumor-associated CD8+ T cells via Treg suppression. PI3K inhibitor-primed tumors become responsive to anti-PD-1 therapy illustrating how cold tumors can be turned into T cell-inflamed ones, responding to ICI (36). Copanlisib use also induced diffused large B cell lymphoma ABC subtype to regress by blocking BCR dependent and independent NF-κB and AKT in an ibrutinib resistant model and combination use results in complete response (37).

Taken together, the use of PI3K inhibitors represents an opportunity to break immune tolerance in the TIME in multiple cancer subtypes.

## 4 Challenges in the use of PI3Kδ inhibitors in the clinic

From clinical trial experiences, a common theme has emerged. PI3Kδ inhibitors induce relatively quick response initially, resulting in improvement in ORR or PFS evaluated as an early clinical trial endpoint. However, in the long term, these responses have not translated into an OS advantage, raising the question of whether the predicative values of ORR/PFS are appropriate clinical endpoints for this class of drug (14). Efficacy in B cell lymphoma has to do with PI3Kδ’s ability to drive transformed B cells out of their protective lymph node or spleen niche which has the protective stroma to provide adhesion, survival or homing signal to CLL cells as evidenced by peripheral blood lymphocytosis initially after the inhibitor use (38, 39) although lymphocytosis is not always evident when combined with rituximab (40).

Evaluating the side effect profile in patients treated with PI3Kδ inhibitors revealed many serious adverse events (AEs), including infections such as pneumonia, and autoimmune AEs such as diarrhea/colitis, hepatitis and pneumonitis which accounted for the fatalities seen in the trial. Indeed, the FDA mandated a box warning in the approval of idelalisib and later for duvelisib, regarding the risk of death due to drug-induced toxicities. Fatal or serious infection happened in 21–48% of patients who received idelalisib and 9–11% for duvelisib per drug insert. The side effects are even more prominent when these drugs were used in untreated populations (40, 41). AEs associated with the use of PI3Kδ inhibitors are related to interruption of signaling mediated by the PI3Kδ. Given the role of PI3Kδ in B and T cell antigen receptor signaling and impaired innate natural killer cells, macrophage function and immune response anticipated in PI3Kδ use, infections such as pneumonia or sepsis are anticipated. This could be attributable to impaired CD4/CD8+ effector cell differentiation, which may lead to infections including bacterial, viral such as cytomegalovirus, or opportunistic like pneumocystis pneumonia. Diarrhea/colitis, pneumonitis and increased liver function tests are autoimmune in nature and have been linked to dysfunction of T-regulatory cells when PI3Kδ is suppressed (17, 42). It is noteworthy that copanlisib, which is considered a pan PI3K inhibitor, characteristically induced hyperglycemia and hypertension due to its effects on the alpha subunit of PI3K. Fortunately, these are transient (43). Analysis of 5 published trials including linperlisib failed to link other specific side effect(s) clearly to the subunit they are preferentially targeting possibly due to the overlapping nature of side effects these other subunits target simultaneously. Serious AEs led to a high rate of dose adjustment (58%) and discontinuation (39%) in clinical trials (44). Infection rates almost doubled from 9.8% per 100 person-years to 18.4% in the treatment group leading to shorter days on drug 173 vs 473 and a numerically higher mortality rate in Medicare beneficiaries receiving idelalisib (45). Obviously, if an infection is not taken care of properly, increased mortality becomes a genuine threat to improving survival. Indeed, patients on trial did well initially but the advantage was erased as the trial continued, with more cumulative incidence of serious AEs. It should be noted that the COVID pandemic may have contributed to less frequent follow up and infection control (6).

**Figure 2:**
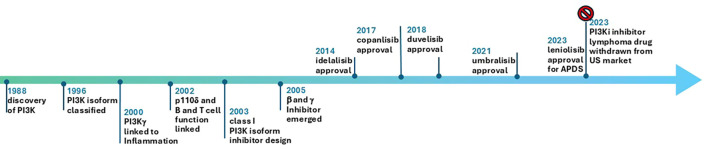
Key events in understanding the biology of PI3K pathway and therapeutic milestones.

As of January 1, 2025, all FDA approved PI3Kδ inhibitor indications for lymphoma have been withdrawn in the US and ten trials for PI3Kδ are recruiting: Roginolisib in melanoma (NCT07203391, NCT06717126), myelofibrosis (NCT06887803), CLL(NCT06644183), R/R peripheral T cell lymphoma (NCT07018752) and non-small cell lung cancer (NCT06879717). HMPL-689 for relapsed or refractory (R/R) lymphoma (NCT05713110), and linperlisib in R/R peripheral T cell lymphoma (NCT06083701; NCT05949944) and R/R cutaneous T cell lymphoma (NCT06037239). Of note, linperlisib received approval in China in 2022 based on a phase 2, single arm study with ORR as the primary end point (46), but it is only available in China. Study of this drug in the US will need solid efficacy and safety data. Further hope for the development of PI3Kδ inhibitors in hematological malignancies and solid tumors has suffered due to FDA withdrawal of approval. The authors suggest that the potential of PI3Kδ inhibitors for overcoming immune tolerance is under explored. It is not clear whether the effects of PI3Kδ inhibitor are mediated solely by enforcing the antitumor immunity in heme malignancy (47). The important events from discovery of PI3K pathway to approval of its many inhibitors is depicted in **Figure 2**. PI3K inhibitors tested in a variety of solid tumors are summarized in **Table 1**.

**Table 1:** PIK3CD and PIK3CG targeted agent in development*.

Generic (code name)	Selectivity	Potency against PIK3C (IC50, nM)				FDA approval/trial including benign disease	Clinical trial (NCT#) example
		α	β	δ	γ		
Copanlisib (BAY 80-6946)	α/β/γ/δ	0.5	3.7	0.7	6.6	Indolent lymphoma Solid tumor	04895579 03842228
Taselisib (GDC-0032)	α/γ	0.29	9.1	0.97	0.12	ST, myeloma, lymphoma	02465060
Eganelisib (IPI-549)	γ	3200	3500	8400	16	TNBC, Bladder	03961698
Duvelisib (IPI-145)	δ	1600	85	2.5	27	Mel, HNSCC	04688658 05057247
Tenalisib (RP6530)	δ	300	100	25	33	ER+ BC	05021900
(AZD8154)	γ/δ	60	1250	6	8	Asthma	03436316
Idelalisib (CAL-101)	δ	820	570	2.5	89	CLL/FL, CRC	05725200 06846671
Umbralisib (TGR1202)	δ	10000	10000	6.2	1400	CLL/MZL	03364231
Nemiralisib (GSK2269557)	δ	5000	1600	0.13	6300	COPD/APDS	02567708
Leniolisib (CDZ173)	δ	240	420	11	2220	APDS, CVID, PID	06897358 06549114
Parsaclisib (INCB50465)	δ	20000	20000	1.1	10000	R/R MCL, FL, ST	02646748 04661007
Seletalisib (UCB5857)	δ	3600	2100	12	280	Sjogren’s psoriasis	
Zandelisib (PWT-143)	δ	5000	210	5	2100	FL, MCL, R/R NHL	04745832
Roginolisib (IOA-244)	δ	19000	2900	150	29000	ST	04328844 06717126 07203391 06879717
Linperlisib (YY20394)	δ	1200	140	4.6	5200	Thymic cancer R/R Lymphoma, PTCL; R/R cutaneous T cell lymphoma	04975061 06083701 05949944 06037239
Amdizalisib (HMPL-689)	δ					R/R Lymphoma	05713110
(AMG319/ACP319)	δ	33000	2700	18	850	R/R NHL, HNSCC	02540928

* completed or ongoing; Abbreviations: PIK3CD, PI3K delta; PIK3CG, PI3K gamma; IC50, 50% activity inhibitory concentration; nM, nanomole; ST, solid tumor; TNBC, Triple negative breast cancer; Mel, Melanoma; HNSCC, Head and neck squamous cell cancer; ER+BC, ER+ Breast cancer; CLL, Chronic lymphocytic leukemia; FL, Follicular lymphoma; CRC, Colorectal cancer; COPD, Chronic obstructive pulmonary disease; APDS, activated PI3K delta syndrome; CVID, Common variable immune deficiency; PID, Primary immune deficiency; MZL, Marginal zone lymphoma; R/R, Relapsed/Refractory; NHL, non-Hodgkins lymphoma PTCL, Peripheral T cell lymphoma; NCT, National clinical trial.

## 5 Lessons learned and ways to move forward

The development of PI3K inhibitors is marked by the initial excitement of high objective response rate and progression-free survival (PFS) targeting diseases that are of indolent nature. However, longer observation failed to establish OS benefit from the treatment intervention and showed potentially harmful effects when these drugs were given on a continuous daily dosing schedule (9, 12, 48). This demonstrates a large gap between early efficacy (tumor-based) measures (ORR, PFS) and long-term survival which has been the gold standard of oncology trials (14). It is in sharp contrast with trials involving ICI in which OS benefits are seen without the ORR and PFS benefit perhaps characteristic of immunotherapy (49, 50). The treatment groups receiving PI3Kδ invariably had higher incidence of SAEs leading to the FDA recognition of this class-specific fatal AE with high rate of toxicity. With only studies of continuous dosing of these agents, with very high cumulative SAE and AEs, the FDA was left with no choice but to withdraw approval.

Activation-induced cytidine deaminase (ACD) is a B cell specific enzyme that targets immunoglobulin genes to initiate class switch recombination and somatic hypermutation. Its activity is tightly regulated by PI3K pathway normally. Chronic PI3Kδ inhibitor use is shown to increase somatic hypermutation and chromosomal translocation. This safety concern is especially relevant if patients stay on PI3K inhibitors long term (51, 52). From a small subcohort study of APDS patients who have constitutively active PI3Kδ pathway (n=9), continuous leniolisib long term (~4.5 years) conferred clinical benefit and seemed to be safe when carefully selected and monitored (53). Several questions remain: 1) Do we need continuous dosing for the agent to be effective? 2) Have we established the minimal effective dose instead of the conventional maximum tolerated dose? 3) Can we mitigate the potentially lethal side effects by simply dosing less frequently? 4) What are the most appropriate areas to further explore PI3K inhibitors in cancer therapy?

### 5.1 Dosing paradigms: optimal biological dose and intermittent administration

Traditionally, dose-finding studies settle somewhere when the majority of patients can tolerate a drug with acceptable toxicity based on maximally tolerated dose (MTD). This strategy is designed for maximum kill of tumor cells without lethal damage to the patients in a period defined as the dose limiting toxicity window. This works well for traditional cytotoxic chemotherapies. Chemotherapy side effects are usually acute in nature and disappear before the next cycle. However, with the emergence of biological response modifiers, the toxicity window becomes unpredictable. This presents a challenge in clinical trial design and in the determination of safe doses in a large patient population. Alternative methods to determine the recommended phase 2 dose have been proposed. Optimal biological dose (OBD) is the most attractive. With OBD, escalation adapts accordingly to account for both efficacy and toxicity (54).

To find the advantage of this dosing scheme over MTD, the author reviewed a total of 87 early-phase trial publications of which 81 were subsequently approved by the FDA as targeted therapies. OBDs were reported for 40% (32/81) of these drugs, which included 19 small molecules and 13 monoclonal antibodies. MTDs were not found in 59% (19/32) of molecules studied. When the OBDs were selected as the recommended phase 2 dose (18/32 molecules), the final FDA-approved doses when OBDs was used was 83% of the drugs whereas only 58% of drug dosing was chosen as the recommended phase 2 dose when MTDs were used (55).

Another way of dosing is metronomic—administration of a cytotoxic agent at lower, less toxic doses at a regular interval (56). This scheme is increasingly used in trial design when biological response modifiers such as antiangiogenic, proteasome or anti-inflammatory drugs are used. The goal here is to preserve the efficacy but avoid increased toxicity (35).

The big question for this class of PI3K inhibitors is will it work? As stated earlier, intermittent copanlisib did work. Indeed, only the low dose (not the high dose) was effective for duvelisib in preclinical work (32). Analysis of idelalisib dosing interruption from three clinical trials found that those who needed dose interruption and dose reduction had better outcomes than those who did not need interruption, suggesting that dose interruption and reduction did not affect efficacy and may have been more efficacious (57). There have been no trial(s) comparing intermittent dosing versus continuous dosing. Clearly more data are needed.

In a recent publication (58), Walsh et al. described stronger suppression of PI3K/AKT/pS6 pathway with dual mTORC and PI3Kδ inhibition in their study of T cells. Importantly, PI3Kδ pathway activity was monitored by quantitating pAKT/pS6 phosphorylation levels in T cells after flow cytometry sorting. Using this biomarker, fine tune of PI3Kδ pathway activity seemed feasible. Use of this biomarker in future exploration of safety and efficacy of PI3Kδ is considered crucial and could be potentially game changing if successfully executed.

Intermittent dosing that shuts off the pathway completely certain number of days in a given cycle or continuous incomplete suppression of PI3Kδ based on AKT/pS6 functional assay can all be attempted to achieve a good risk benefit ratio for infection control. The risk of infection (viral, fungal or bacterial) should ideally reach a level that is comparable to those who are not getting PI3Kδ inhibitor with closely CMV monitoring and PJP prophylaxis.

### 5.2 Antibiotics prophylaxis

Infection has been a concern from the beginning when this class of drug was tested. A recent study showed cumulated incidence of serious infection (G3 or above, pneumonia, neutropenic fever, sepsis) was 30.89% (95% CI: 24.33–37.85) compared to BTK inhibitors with 19.86% (59). These infections could be in the form of bacterial, fungal, virus and possibly pneumocystis pneumonia. Antibiotics prophylaxis with levofloxacin, azithromycin, and cotrimoxazole for pneumocystis pneumonia could be useful in these patients on long-term treatment in future exploration. Population of patients targeted for short term antibiotics prophylaxis may include senior citizen (e.g., >=75 years), high risk for pneumonia (e.g., features of bronchial obstruction) or current heavy smokers with COPD during PI3K inhibition. There is always concern for antibiotics resistance when used long term.

### 5.3 Future areas of research exploration targeting PI3Kδ in solid tumors

Intermittent dosing of PI3Kδ inhibitors would allow for new avenues of exploration of this class of drugs. Importantly, this could provide the creation of a TIME that is more favorable for antitumor immunity especially in combination with immune checkpoint inhibitors (ICI), an area of intense focus in clinical trials using other agents. Combination use of PI3K inhibitors with immunotherapy or chemotherapy in solid tumors is under-explored. PI3K inhibitors could be used to address resistance to ICI in immunogenic tumors, with low or no expression of PD-L1. Tumors with low or no PD-L1 expression is clearly a priority in comparison with MSI high colorectal cancer which is enjoying higher success rate with either PD-1 inhibitor or dual PD-1 and CTLA-4 inhibitors. Indeed, the author’s own clinical trial (NCT04895579) attempts to address “the low or no benefit reality” non-small cell lung cancer get from single agent PD-L1 inhibitor durvalumab consolidation after concurrent chemoradiation therapy if their cancer express low or no PD-L1. It could also be used in priming the cold tumor, such as prostate cancer, to immunogenic response to benefit from treatment with ICI.

Metastatic solid tumors are expected to encounter the most resistance in TIME, hence this appears to be the most appropriate setting in which to study these agents. Given the heightened toxicity profile of PI3K inhibitors shown in individuals who have not received treatment for their CLL or carcinoma of the head and neck (40, 41), front line or neoadjuvant settings would be at very high risk to experience unacceptable toxicities and are not recommended.

A PI3K inhibitor with a different mechanism of action has also emerged. Roginolisib (IOA-244), a non-ATP-competitive PI3Kδ inhibitor, was reported to inhibit regulatory T cell proliferation while having limited antiproliferative effects on conventional CD4+ T cells and no effect on CD8+ T cells. In colorectal and Lewis lung carcinoma models, roginolisib sensitized the tumors to αPD-1 and favored the infiltration of CD8 and natural killer cells, while decreasing suppressive immune cells (60). Roginolisib is currently in clinical phase Ib/II in solid and hematologic tumors (NCT04328844 and NCT06644183) and other solid tumor such as melanoma and non-small cell lung cancer (**Table 1**).

## 6 Concluding remarks

Nearly 30 years after the discovery of the *PI3Kδ* gene, targeting PI3Kδ signaling proved to be a safe and feasible for B cell malignancies. In addition, it has drawn attention to translational researchers as a promising entry point for modulation of the TIME to favor cytotoxic tumor killing by T lymphocytes. However, treating this kinase as a simple on and off switch has been met with excessive and lethal AEs including death. Promising drugs were left with unproven efficacy due to toxicity and withdrawal from the market, leaving many outstanding concepts yet to be tested in solid tumors. Although clinic setbacks have led to the withdrawal of PI3K inhibitors, current evidence suggests that these are temporary challenges rather than a definitive endpoint for this therapeutic approach. Significant changes in the way we drug patients may be necessary, however, and further exploration of intermittent dosing or metronomic dosing should be considered by the scientific community.
